# Systematic review with qualitative meta-synthesis of parents’ experiences and needs in relation to having a child or young person with a mental health difficulty

**DOI:** 10.1136/bmjment-2024-301518

**Published:** 2025-03-25

**Authors:** Faith Martin, Dania Dahmash, Sarah Wicker, Sarah Glover, Charlie Duncan, Andrea Anastassiou, Lucy Docherty, Sarah Halligan

**Affiliations:** 1 Department of Psychology, University of Bath, Bath, UK; 2 Coventry University, Coventry, UK; 3 Cardiff University, Cardiff, UK; 4 Parental Minds, Honiton, UK; 5 British Association for Counselling and Psychotherapy, Lutterworth, UK; 6 University of Bath, Bath, UK

**Keywords:** adult psychiatry, child & adolescent psychiatry

## Abstract

**Question:**

What are the experiences and needs of parents of children and young people (CYP) aged 5–18 with diagnosed mental health difficulties, particularly in relation to the parents’ own well-being?

**Study selection and analysis:**

A systematic review with thematic meta-synthesis was conducted, including qualitative studies published in English. Seven databases were searched (MEDLINE, PsycINFO, CINAHL Ultimate, AMED, EMBASE, Web of Science and Cochrane Library) from inception to September 2024. Studies focused on parents of CYP aged 5–18 years, where the CYP had a confirmed mental health diagnosis.

**Findings:**

Of 75 862 screened studies, 46 met inclusion criteria. Six overarching themes were identified: support needs and gaps; impact on everyday life; altered family dynamics; parental worries and fears; emotional experience of caregivers and self-care paradox. Parents face significant challenges, including unmet support needs from healthcare and education systems, substantial impacts on daily life and altered family dynamics. Emotional experiences such as worry, guilt and stigma were pervasive, compounded by systemic gaps in information and resources. Parents often prioritise their child’s needs over their own, creating barriers to self-care. These challenges were consistent across diagnoses but heightened in cases of life-threatening conditions like eating disorders and depression.

**Conclusions:**

The findings highlight support needs for parents of CYP with mental health difficulties. Tailored interventions, better professional training and family centred care are needed. Future research should focus on developing theoretical models of parental distress to guide interventions and inform support mechanisms that mitigate these broad impacts on parents’ well-being.

**WHAT IS ALREADY KNOWN ON THIS TOPIC**

- Previous research has highlighted the significant psychological and practical burdens faced by parents of children with additional needs, but there has been little focus on synthesising qualitative evidence about the experiences of parents of children with formal mental health diagnoses.

**WHAT THIS STUDY ADDS**

- This study provides the first comprehensive synthesis of qualitative evidence capturing parents’ subjective experiences across a range of child mental health conditions, highlighting commonalities such as emotional distress, unmet support needs and barriers to self-care, alongside diagnosis-specific challenges.
- This study highlights the importance of considering the parents’ own development.

**HOW THIS STUDY MIGHT AFFECT RESEARCH, PRACTICE OR POLICY**

- This study provides detailed evidence of parents’ needs and suggestions for future interventions.

## Background

 The majority of mental health problems initially emerge before the age of 25 years.^1^ In the UK alone, around 1 250 000 children aged 5–19 years have a diagnosable mental illness, with Child and Adolescent Mental Health Services (CAMHS) having the capacity to work with only about one-third.^2^ Following COVID-19, there is an ever-greater demand for CAMHS,^3^ with more parents waiting for CAMHS and trying to support their child themselves. (‘Parents’ is used for any adult in the parenting role.)

The mental health of children/young people (CYP) is linked to parents’ mental health,^4^ owing to reciprocal impacts and interdependency in families.^5^ Significant evidence illustrates the impact of parents’ mental health on CYP.^6 7^ Equally, having a child with a mental health difficulty can adversely affect parents, impacting their well-being, stress and child/family relationships.^8^ Greater depression and stress are reported by parents of CYP with a mental health diagnosis compared with those without.^9^ This can lead to increased service use by parents for their own psychological well-being and lost productivity through time off work.^10^ Support for parents where their CYP has a mental health difficulty is therefore critical to achieving better outcomes for affected families. Nonetheless, a recent scoping review focused on parental involvement in interventions for CYP with anxiety and depression highlighted significant heterogeneity in approaches to supporting or involving parents, and a lack of clarity in conceptualisation of parents’ needs.^11^ Given calls within practice to provide ‘whole family’ approaches and greater support for parents,^12 13^ a comprehensive understanding of parents’ lived experiences of having a CYP with a mental health difficulty is essential to conceptualise their needs and inform future intervention development.

## Objective

To synthesise the current evidence, focusing on qualitative papers, to address the overall research question: what are parents’ experiences of having a child with a mental health difficulty?

## Study selection and analysis

This is a systematic review of qualitative papers. Our published protocol covered both qualitative and quantitative studies,^14^ with quantitative findings being reported separately.^9^

### Search strategy and selection criteria

MEDLINE, PsycINFO, CINAHL Ultimate, AMED, EMBASE, Web of Science and Cochrane Library (including Cochrane Database of Systematic Reviews, Cochrane Central Register of Controlled Trials, Database of Abstracts of Reviews of Effects, Health Technology Assessment Database and NHS Economic Evaluation Database) were searched by FM, from database inception until 18 September 2024. Boolean operators were used to combine terms related to parents; children and young people; mental health difficulties and needs, experiences, well-being for parents (online supplemental material 1). Searches and study selection were done according to the Preferred Reporting Items for Systematic Reviews and Meta-Analyses (PRISMA).^15^ Reference lists of included papers were also manually checked.

Inclusion/Exclusion criteria underwent review by a patient public involvement (PPI) group. Input from the PPI group was facilitated by our PPI lead (SG), who recruited interested parents via a peer-support organisation for parents of children with mental health difficulties. To maximise opportunities for involvement, we provided short presentations and discussions in online meetings, including an orientation to the purpose and methods of a literature review, as well as emailed information and receipt of emailed comments, particularly important for those who could not attend scheduled meetings owing to childcare priorities. The information provided was used in discussions within the study team prior to finalising the literature review protocol. Studies were eligible if they were: published in English or French; included qualitative examination of the experiences of parents or the impact on parents of having a CYP aged between 5 and 18 years with a mental health difficulty, with a majority of CYP in the study falling within this age range; included parents of CYP formally diagnosed with one or more of the following mental health difficulties: depression, anxiety disorders, psychoses, oppositional defiant and other externalising disorders, labels of emerging personality disorders, eating disorders and attention deficit (hyperactive) disorders. Following the presentation of options for exclusion criteria, the advice of the PPI group was used to create the final decision. Studies were excluded if they focused solely on special education needs, for example, autism spectrum conditions; included only parents of CYP who self-harm, as these are the subject of another review^16^ or focused solely on post-traumatic stress disorder, as the potential for shared trauma between CYP and parent means experiences and needs may be different.^17^ Additionally, studies related to attention deficit hyperactivity disorder (ADHD) published before April 2015 were excluded, due to the identification of a number of existing systematic reviews that covered papers related to ADHD prior to this point.^18^,^20^ The findings from these systematic reviews are compared with our findings in the discussion.

At least two reviewers independently screened all titles and abstracts, followed by full-text screening by at least two reviewers (FM, DD, AA, LD). Any disagreements were resolved via a third reviewer. The data were managed using Rayyan.^21^

### Data analysis

For each study, data were extracted by one reviewer, checked by another, with discrepancies resolved through discussion with a third reviewer where necessary (DD, FM, SW, LD). Data were extracted into Excel using a standard form, including: author name, date of publication, country, study design, setting, study aim, parent sample, sample characteristics (including the age and the ethnicity of parent and their CYP characteristics). Results sections describing parental experiences of CYP mental disorders were extracted into NVivo for analysis.^22^

Results were synthesised by FM and DD, with initial themes discussed with our PPI lead (SG) and PPI group. Enhancing transparency in reporting, the synthesis of quality research guidelines^23^ and PRISMA were followed (online supplemental material 2,3). thematic meta-synthesis was conducted, as this allows inductive analysis to interpret findings from studies using different methodologies. This is important given the range of study types and our aim to understand parents’ experiences.^24^ Other approaches require the application of existing frameworks or seek to translate concepts across studies, less aligned with our research question.^25^ The process included line-by-line coding of the results sections from all included studies, to identify key concepts. From these codes, descriptive themes were developed, grouping codes of similar meaning. Next, analytical themes were developed to bring together and analyse descriptive themes into more explanatory themes.^24^ Initial analysis and themes were discussed with interest holders (including parents with lived experience from our PPI group, mental health practitioners and expert academics) to develop final themes. Data were examined to explore differences/similarities by CYP diagnosis. Unless specified in the results, themes were observed consistently irrespective of CYP diagnosis.

The quality of each individual study was appraised using the Joanne Briggs Institute Checklist for Qualitative Research.^26^ Studies were assessed by two reviewers independently; any disagreements were discussed and resolved. No difference in attention or weight was given to studies in the synthesis in relation to the quality appraisal.

### Findings

**Figure 1:**
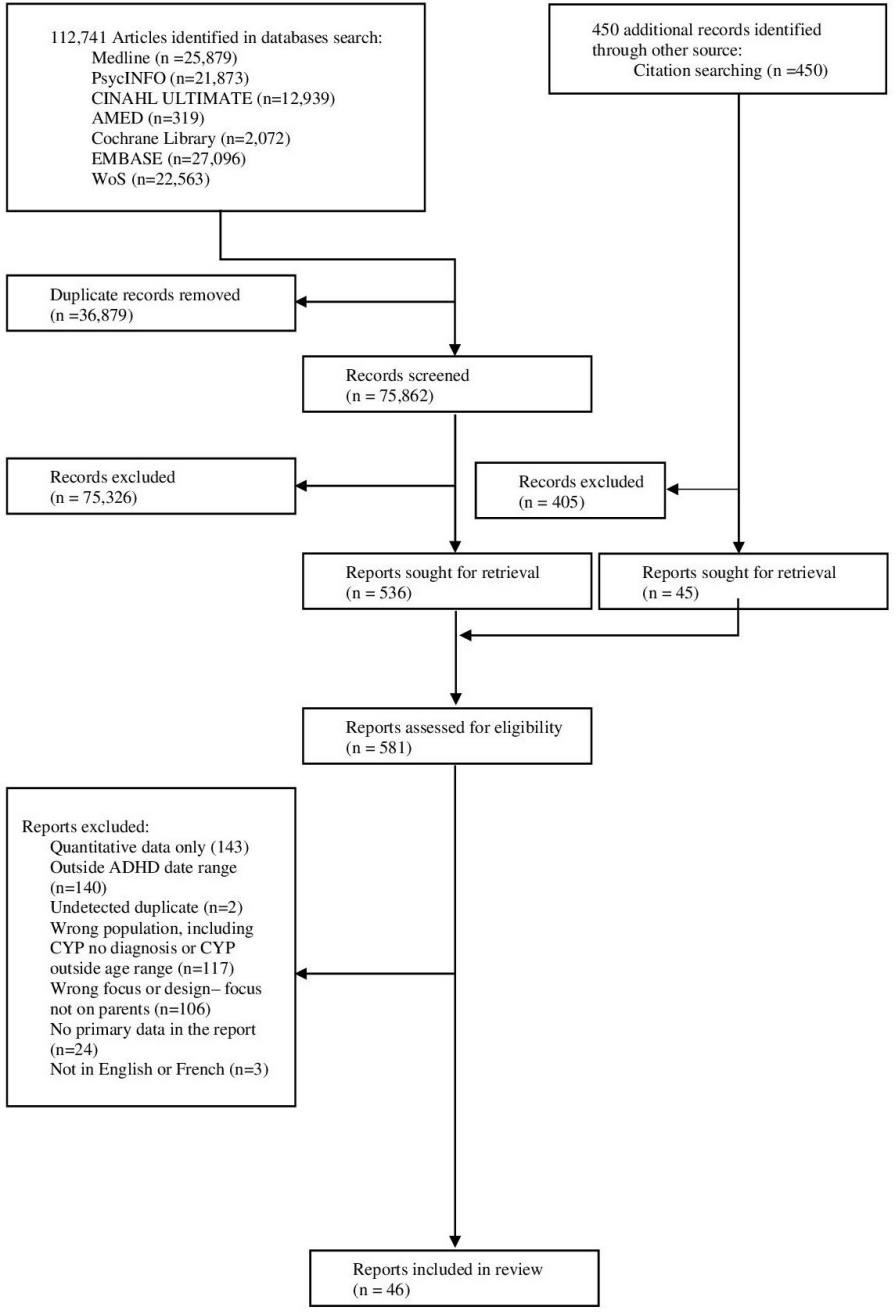
Selection of studies. ADHD, attention deficit hyperactivity disorder; CYP, children and young people; WoS, Web of Science.

Database searching identified 112 741 records. Removal of duplicates led to 75 862 records, with 536 reports sought for full-text screening, leading to the inclusion of 46 studies. Backward citation searching of included studies led to the screening of 450 records, none of which added to the included studies (figure 1).

Included studies focused on parents of CYP with ADHD alone (k=16/46), ADHD or depression (k=1); eating disorders (k=11), depression (k=5), anxiety and depression (k=1); anxiety (k=2), obsessive compulsive disorder (OCD) (k=2), mixed mental health conditions (k=6) and schizophrenia or bipolar (k=2). The majority were from the UK (16/46), the USA (9) or Australia (6). Others were from China (5), Sweden (2), Ireland (2), Turkey (2) and one each from Ethiopia, Tanzania, Israel and Palestine. Typically, semi-structured interviews were used, with thematic analysis dominant. Mean parent ages, where reported, ranged from 21 to 60, with the majority in the mid-late 40s. Five studies were conducted with Chinese populations in China, two with African participants (studies in Ethiopia and Tanzania), one with the ‘ultra-orthodox Jewish’ (study in Israel) community, one with participants described as from the ‘Middle East’ (study in Palestine) and one specifically with ‘African-American’ parents.^27^ Details were not always provided for parents’ ethnicity; however, overall, the majority of studies described a predominantly White/Caucasian sample, often with no further details. Study characteristics are summarised in table 1.

**Table 1:** Study characteristics

Study	Country	Age of CYP (years)	Age of parents (years)	N	CYP mental health difficulty	Setting	Data collection method used	Analysis approach
Ahmann^67^	USA	Not reported	Not reported	4	Mixed	Not reported	Semi-structured interviews	Not reported
Allan *et al*^28^	Australia	5–11	Mean age 43	14	ADHD	Parents who participated in RCT play-based intervention	Semi-structured interviews	Thematic analysis
Armitage *et al*^53^	UK	13–18	Not reported	8	Depression	Outpatient	Semi-structured interviews	Phenomenological analysis
Ay and Doğan^29^	Turkey	12–18	29–51	10	ADHD	outpatient	Semi-structured interviews	Phenomenological analysis
Bai *et al*^78^	China	13–18	38–47	13	Schizophrenia	Inpatient	Semi-structured interviews	Thematic analysis
Bezance and Holliday^66^	UK	13–16	40–63	9	Anorexia	Outpatient	Semi-structured interviews	Phenomenological analysis
Budman and Maeir^30^	Israel	6–18	30–45	10	ADHD	Outpatient	Semi-structured interviews	Thematic analysis
Chan and Mo^44^	China	7–10	Not reported	18	ADHD	Outpatient	Interviews	Thematic analysis
Chessell *et al*^31^	UK	8–14	Mean 44.6	22	OCD	Outpatient	Semi-structured interviews	Thematic analysis
Ching'oma *et al*^32^	Tanzania	6–15	30–over 40	16	ADHD	Records	Semi-structured interviews	Content analysis
Cifra *et al*^54^*	USA	<18	Not reported	14	Eating disorder	Outpatient	Semi-structured interviews	Immersion crystallisation
Cottee-Lane *et al*^46^	UK	13–16	40–65	11	Anorexia	Outpatient	Semi-structured interviews	Phenomenological analysis
Davey *et al*^47^	UK	7–12	Not reported	11	Anxiety	Outpatient	Interviews	Thematic analysis
Eaton *et al*^70^	Australia	5–13	30–56	12	Mixed	Community	Semi-structured interviews	Descriptive phenomenological analysis
Emerson^56^	USA	Mean 14	28–60	10	ADHD	Outpatient	Interviews	Phenomenological analysis
Harazni and Alkaissi^57^	Palestine	7–10	Not reported	4	ADHD	Outpatient	Structured interviews	Phenomenological analysis
Harden^43^	UK	13–16	Not reported	25	Mixed	Via hospital	Semi-structured interviews	Interpretive inductive
Hellerova *et al*^45^	USA	6–17	30–over 50	20	Depression	Outpatient	Semi-structured interviews	Content
Hiscock *et al*^58^	Australia	Mean 14	Not reported	70	Anxiety and depression	Outpatient	Semi-structured interviews	Thematic analysis
Honey *et al*^33^	Australia	14–20	Not reported	24	Anorexia	Outpatient	Interviews	Content analysis
Hunter^59^	USA	10–19	Not reported	4	Eating disorders	Outpatient	Semi-structured interviews	Giorgi’s model
Klein *et al*^34^	UK	0–19	Not reported	13	ADHD	CAMHS	Semi-structured interviews	Thematic analysis
Konstantellou *et al*^35^	UK	14–16	Mean 49.3	17	Eating disorder	Outpatient	Focus group	Phenomenological analysis
Leitch *et al*^60^	Australia	5–12	38.4–50.4	13	ADHD	Records	Focus group	Thematic analysis
Long^52^	UK	5–18	35–42	7	ADHD	Outpatient	Interview	Phenomenological analysis
McArdle^36^	Ireland	12–21	Mean 49.8	15	Eating disorders	Community	Focus group or individual semi-structured interviews	Thematic analysis
McKeague *et al*^61^	Ireland	10–16	21–60	40	ADHD or depression	CAMHS	Semi-structured interviews	Thematic analysis
Mesfin and Habtamu^71^	Ethiopia	Not reported	27–48		ADHD	Outpatient	Interviews and focus groups	Phenomenological analysis
Ott^37^	USA	9–19	Not reported	12	Anorexia	Community	Semi-structured interviews	Consensual
Patel *et al*^38^	USA	Children	Not reported	19	Eating disorders	Outpatient	Semi-structured interviews and focus groups	Constant comparative method
Reardon *et al*^39^	UK	7–11	Mean 43.5	16	Anxiety	Schools	Semi-structured interviews	Thematic analysis
Ringer *et al*^62^	Sweden	6–13	Not reported	12	ADHD	Schools	Semi-structured interviews	Content analysis
Rosenzweig *et al*^55^	USA	Mean 14.7	Mean 44.8	41	Mixed	Community	Focus groups	Content analysis
Ruuskanen *et al*^50^	Australia	7–11	38–50	13	ADHD	Database	Focus groups	Thematic analysis
Saulsberry *et al*^27^	USA	3–18	Mean 40.7		ADHD	Outpatient.	Focus groups	Content analysis
Sheng *et al*^68^	China	12–18	33–49 (mean 40.5)	14	Mixed	Inpatient	Semi-structured interviews	Thematic analysis
Slowik *et al*^63^	UK	NR	Not reported	10	Mixed	Inpatient	Open group	Content analysis
Sowden *et al*^40^	UK	8–18	Mean 47.3	20	OCD	Outpatient and community	Interviews	Framework approach
Stapley *et al*^48^	UK	11–17	32–64	48	Depression	CAMHS	Semi-structured interviews	Thematic analysis
Stapley *et al*^64^	UK	11–17	33–64	85	Depression	CAMHS	Semi-structured interviews	Ideal-type analysis
Svensson *et al*^49^	Sweden	16–18	Not reported	10	Eating disorder	Outpatient	Semi-structured interviews	Phenomenological analysis
Tarver *et al*^41^	UK	6–10	29–52	12	ADHD	Community and outpatient	Semi-structured interviews	Thematic analysis
Thomson *et al*^51^	UK	11–18	30–59	8	Anorexia	CAMHS	Semi-structured interviews	Phenomenological analysis
Yurdakul *et al*^65^	Turkey	7–12 (mean 9.5)	Not reported		ADHD	Outpatient	Semi-structured interviews	Phenomenological analysis
Zhang *et al*^42^	China	12–18	38–52	14	Depression	Inpatient	Interviews	Phenomenological analysis
Zhang *et al*^69^	China	5–13 (mean 8.9)	29–50 (mean 38.2)	20	Schizophrenia or bipolar	Inpatient	Interviews	Phenomenological analysis

* The study is an abstract.

ADHDattention deficit hyperactivity disorderCAMHSChild and Adolescent Mental Health ServiceCYPchildren and young peopleOCDobsessive compulsive disorder

Critical appraisal of the studies revealed 30% (14/46) had evidence of all quality criteria, with 83% meeting eight or more of the 10 criteria (online supplemental material 4). Where quality was lower, this typically related to elements of reflexivity, including the researcher’s acknowledgement of their culture or theoretical position and influence on the research. Analyses typically focused on establishing the fundamental nature of the parents’ experiences, often with limited links to applied theory either in the analysis or within the discussion.

Parents’ experiences of their CYP’s mental health difficulties were characterised by six themes: (1) support needs and gap; (2) impact on everyday life; (3) altered family dynamics; (4) parental worries and fears; (5) emotional experience of caregivers; (6) self-care paradox. (The appearance of themes in each included study are summarised in online supplemental material 5). Quotes illustrating each theme are provided in table 2.

**Table 2:** Quotes from included studies to illustrate each theme

Theme	Subtheme	Quotes
1. Support needs and gaps	Communication challenges in support from healthcare	“At times, I’ve felt like I’ve been, had my wrists slapped for accommodating things, which hasn’t felt very nice. it was quite a distressing moment”.^40^
	Uncertainty and concerns around treatment	*“What will happen next that worries me? Until when will the drug be used?”* ^29^
	Communication with schools driven by crisis	*“the school’s goal the whole time has been to try to be able to handle it, and when they didn’t feel like it was working anymore they spoke up…there were quiet periods and then here comes a huge problem”.* ^62^
	Need for information	*“When you’re given a diagnosis that’s just face-to-face, you just hear the diagnosis, you don’t hear anything else about it”* ^47^ *“People kept saying to me you need more support. Where?”* ^46^
2. Impact on everyday life	The demands of caring for a child with mental health difficulties	*“negotiate between their parenting values, everyday demands and [CYP diagnosis*]*”.*^62^*“I had to stay at the hospital everyday. I was too tired to do anything else”.*^68^
	Overwhelming nature of child’s needs	*“*[*taking] over [their] life”.*^66^*“Everything must always be as he says”.*^65^
	Gendered impact on mothers	*‘a lot of these things happened, kind of before school. so it was affecting [partner’s name] a lot more than me because I had already gone to work by then’*.^31^
	OCD: financial and time costs	*“extra shopping, cleaning, washing, purchasing special food or household items, disturbed sleep resulting in an immense disruption to family life”*.^40^
3. Altered family dynamics	Conflict with child	*“Sometimes I feel much stressed and angry when I see that she cannot do anything properly, I hit her and after that I feel guilty”.* ^57^ *“Well, very frustrating, very frustrating not to reach [her], not being up to it, to always finish second in every race, to not be enough, yes, frustrating”.* ^14^
	Strain on marital and family relationships	*“I quarrel with my husband sometimes. It caused family discord”*.^78^*“[M]y family is in a bit of a crisis and the wheels are about to fall off”.*^50^
	New role in monitoring and management of symptoms	*“he would just sit there to monitor his son’s food consumption”.* ^59^
	Changing parenting style to avoid conflict	*“self-monitoring to avoid triggering outburst”.* ^60^
	OCD specific: confusion managing parenting versus managing OCD	*“the confusing nature of OCD”* ^49 66^ *“Defining the boundaries between caring and accommodating was challenging^"^* ^57^
4. Parental worries and fears	Immediate worries about the child’s life	*“anx[ious] for the future”*.^66^*“In the short term, thy worried about their child making friends or academic success”*^32^
	Long-term concerns around stigma and shame	*‘I want to protect his hono[u]r, mainly for the shidduch (marriage match)’.* ^30^ *“I don’t want them to be tagged for the rest of their lives”.* ^61^
	Fear of death	Eating disorder: *“The doctor who was on duty had actually called us into the sister’s office and she said you know this child is extremely ill she may die., which really knocked us for six. It was very, very traumatic”*.^46^Depression: *“Worrying but you know, it’s stupid things like is she going to be alive when I knock on her door in the morning”*.
5. Emotional experience of caregivers	Emotional exhaustion in the face of extreme challenges	*“it totally exhausted us, and stretched us to the limit… For us, it was a real emergency”*.^58^
	Blame from others	*‘‘I felt that she [professional] was blaming me, you know that my child had an eating disorder because of me’’*.^36^
	Self-blame, guilt and sense of failure	“It hurt me so much to be unable to help my daughter. I felt alone, and I was so frustrated!”^73^*Oh I felt terrible. I thought you know how have I let her get like this but you know it was really difficult (cries)’*.^51^*“‘am I a good enough parent?’’(Jane), ‘‘did I do the wrong thing?’’ (Diane), ‘‘am I doing the right thing?’’ (Dee), ‘‘am I enough for him (son*)*?’’ (Jane)”.*^70^*“if I don’t prepare her properly then I failed in my role”*.^30^*“If I were a normal parent who didn’t have ADHD, maybe I wouldn’t feel so overwhelmed with everything”*.^52^
	Cultural stigmatisation and shame	*“They see it as, I wouldn’t say a sign of weakness, they just see it as, just get on with it. it’s not really spoken about”*.^31^*“He would scream in the room and try to jump off the building….I have never been so humiliated in all my life!”*^69^
	ADHD: frustration with accessibility of support	*“ADHD was not seen as a “serious disorder”*.^60^
	Depression and eating disorder: grief and loss of imagined future	*“Where’s my daughter gone because that’s not her”* ^53^
6. Self-care paradox	Prioritising the child’s well-being over one’s own	*“I don’t think as a mother you think about yourself. You think about your daughter”*.^66^
	Feeling guilty or unworthy of self-care	*“It [self-care] was recommended to me but I never did it. I felt like I couldn’t focus on me until I knew she was okay, that she was in an okay place. I couldn’t focus on me at all”.* ^38^
	Caring for your own physical needs	*“if there is some time then I need some rest and to sleep or eat”*.^30^
	Spiritual well-being	*“Prayer is the one thing that calms me down”*.^71^
	Self-care as moments of normalcy	*“So I do kind of quite like it when I do something normal”* ^35^
	Desire for peer support	*“it’s not normal [laughs], so yeah, it’s, it’s embarrassing, it’s secretive”*.^31^*I would like to speak to other people who experience it, the same, or similar sort of things.as a parent, it is quite frightening, yeah, it is very frightening”*.^40^

ADHDattention deficit hyperactivity disorderOCDobsessive compulsive disorder

### Support needs and gaps

Good communication from healthcare services was described as crucial for parents, across CYP diagnoses.^28^,^42^ They expected clear communication,^35 40^ particularly regarding treatment options. Lack of communication and consideration led to resentment of clinicians.^33^ Parents described feeling disregarded, and that the professionals were unaware of their suffering.^43^ Parents felt they were considered only during crises,^40^ but needed support throughout.

A particular need parents described was for information, particularly linked to the challenge of distinguishing ‘normal’ behaviour in relation to CYP development from mental health problems.^31 44 45^ Information desired was specific to CYP diagnosis and where to find support.^2930 32 34 39 40 46^,^50^ They experienced information overload at the point of diagnosis, followed by a lack of information.^47^ Parents actively sought information in different modes: books, websites or television.^46 48^ There was the risk of misinformation^40^ and distress,^51^ warranting provision from professionals.^29^

With respect to differences by CYP diagnosis, healthcare practitioner communication was reported as particularly important for eating disorders, anxiety and OCD,^31 33 36 38 40 47 51^ with concerns about judgement from clinicians.^33^ Parents wanted communication that empowered themselves and their child,^47^ validated their distress and enabled them to support or co-deliver treatment for their child.^36^ Similarly, parents experienced a lack of validation and empathy from schools, instead experiencing judgement and again finding that communication only occurred during crises. For parents of CYP with OCD particularly, the schools’ lack of knowledge was described, requiring parents to coordinate education with healthcare.^31 40^ Some parents of CYP with ADHD reported schools not taking the difficulty seriously,^52^ or less support for male children.^30 37^ Parents of CYP with anxiety disorders and OCD described how their children’s difficulties sometimes appeared irrational, requiring more psychoeducation to understand.^40 47^ Parents wanted resources for themselves, describing the relevant mental health problems and common comorbidities, such as OCD with autism.^31 40 47^ Parents of children with ADHD were often well informed about ADHD ‘symptoms’, but wanted better understanding of how to control symptoms and strategies to support their parenting self-efficacy.^27 28 32 34 41^

### Impact on everyday life

There were significant demands on parents, often linked to real-world barriers to accessing care for their child: scarcity of services, long waiting lists and long journeys.^28 31 36 40^ These then impacted time available for activities other than supporting their child, including socialising and household chores. Parents described the overwhelming nature of both their child’s needs and trying to access treatment as draining their energy and resources,^40^ at times leading to depression and anxiety.^53^ Financial strain, due to loss or resignation from job, medical bills and poor insurance coverage, was also frequently described.^32 36 45 54^ One study focused on the impact on working parents, noting the stress inherent in this and a lack of statutory support.^55^ Study parents adopted strategies from flexibility and compromises to exercising ‘military routine’ to manage the multiple demands. The gendered impact was noted, with typically a greater demand on mothers.^31^ Impacts on everyday life were highly related to individual circumstances, rather than CYP diagnosis, with the exception of the cost and time linked to compulsions for parents of CYP with OCD.

### Altered family dynamics

Parents described altered relationships and roles, with their CYP and wider family.^2729 32 38 39 44 49^,^51 53 56^ Conflict with their child was common,^65^ often arising from misunderstandings of their child’s behaviours, creating a vicious cycle. This led CYP, feeling rejected, to respond with hostility, deepening conflict. At times, parents described trying their best but being perceived by CYP as not understanding.^48^ Parents expressed high frustration and exhaustion. Physical punishment was their last resort to recover control, leading to shame and guilt.^29 32^

Conflict between parents was observed, with some feeling judged or judging their spouse/partner’s responses.^31^ With parents being highly focused on their child’s mental health needs, sometimes other children in the family felt neglected or resentful, leaving parents distressed.^56 60^

Within the altered family dynamics, there was a shift in parents’ roles. Parents became monitors/observers,^60^ care coordinators and trainers of others and advocates.^27 40 43 60^ Parents monitored behaviour in themselves, their CYP and others to avoid triggering an outburst. For potentially life-threatening difficulties, for example, eating disorders and depression, parents were more attentive to their children’s symptoms and behaviour, protecting to the point of becoming overbearing.^59^ Parents talked about feeling helpless in these roles, deepened by a lack of support from professionals.^43^ Furthermore, parents of CYP with eating disorders and OCD may be asked to support, co-provide or lead therapy. Many expressed difficulty with this, owing to their own emotional state. Some felt they lacked confidence to make required decisions.^39^ Parents of children with OCD described not trusting their judgement, owing to the risk of inadvertently perpetuating their child’s symptoms.^31 40^ These role changes were enacted by a subjugation of parents’ own needs.^30^

### Parental worries and fears

Parents expressed a range of worries, particularly anxiety about the immediate future, including topics of making friends, academic success, chances to marry and long-term concerns, particularly focused on stigma linked to being labelled with a mental health condition.^32 47 66^ Parents expressed worries about safety and relapses.^49 53 57 67 68^ These thoughts were frequent and disruptive: *“You wake up thinking about it and checking your phone in breaks at work and thinking is she alright”*.^53^ Owing to risks associated with eating disorders and depression, these parents discussed their child’s future with gravity. Parents feared the death of their child, by eating disorder or suicide and felt powerlessness to protect their child.^49^ Some parents described trauma from the realisation of the risk and the life-threatening nature of their child’s difficulties.

### Emotional experience of caregivers

Parents’ emotional experiences extended beyond worry. Acute emotional distress was evident in parents’ vocabulary and expression: *‘overwhelmed’, ‘exhausted’*, *‘draining’, ‘living nightmare’*.^46 48 56 58^ Parents experienced insomnia and felt paralysed.^29 56^

Parents described how society perceived their CYP’s mental health problems, judging them and lacking sensitivity.^30 37^ Many described feeling blamed, guilt and shame.^42 62^ Blame was common, from healthcare, school, other parents, family and partners.^4857 61^,^63^ In addition to stigma from others,^69^ some parents described internalised stigma about having a CYP with a mental health problem, increasing their sense of responsibility to fix distress.^42^ Parents expressed blaming themselves and doubting their aptitude as caregivers.^53 70^ They felt powerless, impacting parenting self-efficacy.^35 57 66^

Parents described a sense of failing their CYP,^43 66^ reducing parenting self-efficacy further and creating significant guilt and tension. The impact of this was significant: most parents described withdrawing from social interaction to protect themselves,^42 51^ which some study participants linked clearly to avoiding others and doubting themselves.^70^ These feelings linked to frustration, with the situation and the lack of priority given to mental health compared with physical health,^40^ and particularly for parents of children with ADHD.^30 61^ In some studies, issues of shame were linked to the cultural meaning of the child’s difficulties as a parents’ failure and not something where support from others is provided.^31 69^ Chronic impacts were seen, particularly with parents of CYP with eating disorders and depression, who described having to process the risk of their CYP’s death, grief and sorrow.^53 63^ This chronic sadness related to the loss of a healthy child and/or of the child’s imagined future, which they feared or now knew would not be possible.^49 53^

### Self-care paradox

Considering self-care, parents described the paradox of reduction in self-care owing to significant other demands, together with a need for greater self-care. Parents across all CYP diagnoses shared that their priority was the well-being of their youth. Some parents mentioned being encouraged to self-care; however, they described how their sense of guilt, shame or embarrassment made this difficult. Parents typically only sought help for themselves and their family due to a crisis.^50^ The focus was on physical needs (sleep, food). Some did recognise that they needed to take a time out,^40^ with evidence of use of existing coping mechanisms, such as prayer for spiritual well-being.^71^ In many cases, parents wanted to do something ‘normal’ to find a break from the stresses.^35^ This was important for the whole family. Parents of children with eating disorders and ADHD diagnosis emphasised the need for peer support and a safe space to share their experiences.^35 38 60^

## Conclusions and clinical implications

The themes highlight the non-trivial impact of CYP mental health on many parents, showing limitations in current support and needs for further information. CYP mental health difficulties had an impact on parents’ everyday life, including work. Their relationships and roles within their families were altered. Specific worries and fears linked to the actual and perceived consequences of their child’s difficulties were common, but emotions for parents extended into guilt, shame, self-blame and sense of failure. Despite all this, many parents found it difficult to take care of their own needs, not least because of the requirement to support their young people, but also due to a sense of not deserving self-care.

Similar themes were seen in reviews of qualitative studies with parents of CYP with ADHD, which reported emotional distress, need for validation and support and impacts on everyday life.^18^,^20^ Here, parents described a significant impact on their sense of self, often with a sense of failure that their child has a mental health difficulty. They also reported changes in work and family relationships. Parents often held a care-coordination role: becoming an expert, sharing information, advocating and managing practical demands. As the adult and carer, the parent is often at the nexus of CYP needs and demands from various systems. The stress of this role was clear, as was the need for better support.

Across different CYP diagnoses, there were some differences. Behavioural differences seen in CYP with ADHD appeared to mean parents wanted more practical support and coordination from the schools than was evident in other conditions. The differences in parents’ experiences related to features of their CYP’s experiences: higher levels of risk to CYP’s physical health and/or life were associated with greater distress, fear for the future and often more impacts on everyday life as parents reduced other roles to be with and care for their CYP. The extent to which parents needed to develop new roles related in part to CYP risk, and to their access to treatment and parents’ involvement in that treatment. The importance of different elements of CYP presentation and family situation requires further exploration to identify the extent to which different interventions are needed to support different parents.

The conflict and challenges to wider relationships may feed back into CYP’s mental health, through the interdependency and circularity within families.^5^ Family conflict is a risk for the development of CYP depression, for example, but it also maintains it.^72^ The parents’ understandably high levels of distress about their CYP’s mental health may increase overall levels of emotional arousal and tension in the family, affecting CYP mental health. Furthermore, parents may not engage in self-care, often due to decreased opportunity and/or shame, meaning they feel self-care is undeserved. Supporting parents in self-care is vital for their well-being, and to model this to their CYP.^73^

As CYP move into and through adolescence, they individuate, with parents’ roles shifting to support independence.^74^ Conversely, here, parents often described increasing monitoring and responsibilities to secure care, and less time for themselves. The socially normal family development is disrupted. Protective processes are important for family resilience, particularly the reduction of risk by creating safety; the interruption of negative cycles through positive interactions; self-efficacy in managing stressors and opportunities for family members to support one another.^5^ Our review highlights risks and challenges to these protective processes, emphasising the need to support parents to be able to use these processes.

Parents’ own development was affected. Where reported, most parents were in the ‘established adulthood’ age range.^75^ Increased caring responsibilities and concern about their CYP placed pressure on parents of this age, who are often also managing work demands and older parents.^75^ Typically, at this stage, adults are developing to feel secure in their roles and identities, gaining satisfaction from romantic relationships and feeling more confident.^76^ Our findings show that these developmental tasks are disrupted for parents of CYP with mental health difficulties. Support for parents then must include consideration of their own development. The findings provide several recommendations for practice, as summarised in table 3.

**Table 3:** Recommendations arising from review findings

Recommendation	Rationale
**Training for staff**Provide healthcare professionals and school staff with brief training related to (a) the experiences of parents of CYP with mental health difficulties and (b) communication training.	Parents’ descriptions of their experiences were highly influenced by healthcare and school staff. Helping staff to understand parents’ common experiences and the significant impact on many parents, across a range of areas of life, including the parents’ own development, all may contribute to staff’s belief in the need to change or maintain good communication and supportive approaches with parents. Continued development and implementation from staff or their communication skills may reduce the negative impacts and increase the positive outcomes of interactions between healthcare/school and parents. Parents connected being informed with reduced distress.
**Information for parents**Provide parents with specific information about where to find support. This can be delivered by healthcare, school and charitable sector. This may be during discussions and appointments, as well as public information on websites. This information should be offered at multiple time points, as the child’s and parents’ needs may change owing to the course of the child’s difficulties.	Parents were advised to seek support; however, often did not know where to find it. They required specific advice, for example, about local peer support groups, adult mental health-focused support or nationally available support via charities such as ‘Young Minds’ and ‘Charlie Waller’ in the UK.
**Develop multiple support offers**To enable useful information about support, there must be an increased provision of support for parents. There should be a range of types and foci for parents’ support. This may include: parenting training approaches to directly impact on skills related to how to support their child, emotionally focused and peer-delivered intervention to tackle guilt and shame and self-care or self-management focused interventions aimed to improve parents’ own emotional state and coping through behavioural change. Family therapy can also address parents’ well-being.	These qualitative findings, coupled with quantitative results from a linked review,^9^ highlight parents’ need for support and the non-trivial nature of the impact on many parents. As parents’ experiences range across different difficulties, with different potential causal mechanisms (eg, low parenting self-efficacy, lack of knowledge, psychological processes related to shame, cognitive and behavioural barriers to self-care), a one-size-fits-all approach to supporting parents is unlikely to meet all needs.
**Develop an underpinning model**Research should focus on the development of a clear, underpinning model of parents’ distress.	This is vital to (a) better target interventions at causal mechanisms most linked to parents’ distress and (b) help identify characteristics linked to the child and parent that are associated with greater risk of parental distress, to allow targeting of intervention.

CYPchildren and young people

### Limitations and future directions

This review relies only on published studies in English. The majority of included studies were conducted in high-income countries, typically with mothers, again limiting the transferability of the findings. Our age-range limit for the CYP was up to 18 years; however, for some conditions, we may have missed literature focusing on young people, potentially still living with their parents. For example, the psychosis literature may have been missed if recruiting from services for CYP aged up to 25 years, as is common. Any qualitative analysis is influenced by the researchers’ position,^77^ meaning others may interpret the data differently. Our research team includes clinicians, academics and people with lived experience, and we sought and incorporated comments on initial analytical themes from academics, practitioners and parents with lived experience. It is a strength of our study that we worked with a PPI group; however, the practical restriction to only online/emailed contact may have inadvertently excluded people owing to the reliance on internet access. The reviewed studies were primarily with White/Caucasian samples, and in English (no relevant articles in French identified). Future reviews should include a broader range of languages. The findings of our review may be limited in transferability to other ethnic and cultural groups, owing to the limitations of the evidence base.

Future research should establish the links and relevant importance of different revealed themes on parents’ well-being. For example, lack of information may relate to fear for the future and reduced parenting self-efficacy, creating worry and blame and guilt. Lack of understanding might relate to conflict with CYP, increasing guilt. Lack of time and prioritisation of oneself may reduce self-care, potentially worsening emotional impacts. However, these links require further elaboration through the building of a model of parents’ distress. For example, is parenting self-efficacy the dominant driver of parental distress, demanding then parenting training? To what extent are cognitive processes related to guilt and shame relevant in parents’ experiences, suggesting self-compassion interventions? Quantitative studies could usefully test such links/models. Further studies with parents of children with conditions other than ADHD are required, as is greater consideration of the parents’ development. Further research including parents from a wider range of ethnic, cultural and socio-economic backgrounds is required.

## Conclusions

In summary, parents of children with mental health difficulties describe significant impact on their lives, with a need for greater support from services, an impact on everyday life, altered relationships and roles, significant emotional distress and challenges to being able to engage in self-care. Research to understand the mechanisms of parents’ distress would inform the development of much-needed interventions to specifically address the impact of CYP mental health difficulties on parents.

## supplementary material

10.1136/bmjment-2024-301518 online supplemental file 1

## Data Availability

Data were drawn from existing, published studies.
