# Supplementary material for: Systematic review with qualitative meta-synthesis of parents’ experiences and needs in relation to having a child or young person with a mental health difficulty
Source: BMJ Ment Health. 2025 Mar 25;28(1):e301518. doi: 10.1136/bmjment-2024-301518 (PMC11950944; doi:10.1136/bmjment-2024-301518)
Supplement: online supplemental file 1 [file bmjment-28-1-s001.docx]

**Supplemental Material 1: Full searches for Medline**

| S1 | TI ((parent or parents or parental or mother or father or care*giver or guardian* or carer* or paternal or maternal) ) OR AB ( ( parent or parents or parental or mother or father or care*giver or guardian* or carer* or paternal or maternal ) ) OR MM ("Parents+") |
| --- | --- |
| S2 | TI ( (children or adolescent* or adolescence or youth* or child or teenager* or pediatric* or paediatric* or kid* or teen* or young person or young people or boy* or girl* or juvenile* ) ) OR AB ( ( children or adolescent* or adolescence or youth* or child or teenager* or pediatric* or paediatric* or kid* or teen* or young person or young people or boy* or girl* or juvenile* ) ) OR MH ("Child+") OR MM ("Adolescent") |
| S3 | ( TI ( ( attention deficit disorder* or “attention deficit hyperactive disorder* “or “ADHD”) OR AB (attention deficit disorder* or “attention deficit hyperactive disorder* “or “ADHD”) OR MH (“Attention Deficit Disorder with Hyperactivity”) OR ( TI ( (Eating disorder* or anorexia or bulimia or eating problem*) ) OR AB ( (Eating disorder* or anorexia or bulimia or eating problem*) OR MH(“Feeding and Eating Disorders+”) OR ( TI ( (Emerging personality disorder* or emerging personality problem*) ) OR AB ( (Emerging personality disorder* or emerging personality problem*) ) OR MH (“Personality Disorder+”) ) OR ( TI ( (Externalising disorder* or externalising problem* or externalizing disorder* or externalizing problem*) OR AB (Externalising disorder* or externalising problem* or externalizing disorder* or externalizing problem*) OR TX (“Externalising disorder”) ) OR ( TI ( (Oppositional defiant disorder* or oppositional defiant problem*) ) OR AB ( (Oppositional defiant disorder* or oppositional defiant problem*) OR (MH“Attention Deficit and Disruptive Behavior Disorders+”) ) OR ( TI ( (Psychos* or psychotic disorder* or psychotic problem*) ) OR AB ( (Psychos* or psychotic disorder* or psychotic problem*) ) OR MH (“Psychotic Disorders+) ) OR ( TI ( ( Anxiety or depression or depressive or “obsessive compulsive disorder” or “OCD” or phobia or phobic or mood disorder or anxiety disorder or panic disorder or agoraphobia or internalising problem* or internalising problem* or internalizing problem* or internalizing disorder* ) ) OR AB ( ( Anxiety or depression or depressive or “obsessive compulsive disorder” or “OCD” or phobia or phobic or mood disorder or anxiety disorder or panic disorder or agoraphobia or internalising problem* or internalising problem* or internalizing problem* or internalizing disorder* ) OR (MH "Depressive Disorder") OR (MH "Depressive Disorder, Major") OR (MH "Depressive Disorder, Treatment-Resistant") OR (MH "Dysthymic Disorder") OR (MM "Anxiety Disorders+") ) |
| S4 | TI (“Information need*” or “knowledge need*” or need* or support or experience*or impact or wellbeing or concern* or want or perspective* or belief* or attitude*or prefer* or anxiety or anxious or depressed or depression or strain or stress or burden or “parent satisfaction” or “family relationship” or “parent* self-efficacy”) OR AB (“Information need*” or “knowledge need*” or need* or support or experience*or impact or wellbeing or concern* or want or perspective* or belief* or attitude*or prefer* or anxiety or anxious or depressed or depression or strain or stress or burden or “parent satisfaction” or “family relationship” or “parent* self-efficacy”)) |
| S5 | S1 N8 S4 |
| S6 | S2 N8 S3 |
| S7 | S5 AND S6 |

**Supplemental Material 2: Enhancing transparency in reporting the synthesis of qualitative research: ENTREQ Checklist**

| **Item No.** | **Guide and Description** | **Report Location** |
| --- | --- | --- |
| 1. Aim | State the research question the synthesis addresses | p4 |
| 2. Synthesis methodology | Identify the synthesis methodology or theoretical framework which underpins the synthesis, and describe the rationale for choice of methodology (e.g. meta- ethnography, thematic synthesis, critical interpretive synthesis, grounded theory synthesis, realist synthesis, meta-aggregation, meta-study, framework synthesis) | p6 |
| 3. Approach to searching | Indicate whether the search was pre-planned (comprehensive search strategies to seek all available studies) or iterative (to seek all available concepts until they theoretical saturation is achieved) | p5 |
| 4. Inclusion criteria | Specify the inclusion/exclusion criteria (e.g. in terms of population, language, year limits, type of publication, study type) | p5 |
| 5. Data sources | Describe the information sources used (e.g. electronic databases (MEDLINE, EMBASE, CINAHL, psycINFO), grey literature databases (digital thesis, policy reports), relevant organisational websites, experts, information specialists, generic web searches (Google Scholar) hand searching, reference lists) and when the searches conducted; provide the rationale for using the data sources | p5 |
| 6. Electronic Search strategy | Describe the literature search (e.g. provide electronic search strategies with population terms, clinical or health topic terms, experiential or social phenomena related terms, filters for qualitative research, and search limits) | Appendix p2 and brief outline p5 manuscript |
| 7. Study screening methods | Describe the process of study screening and sifting (e.g. title, abstract and full text review, number of independent reviewers who screened studies) | pp5-6 |
| 8. Study characteristics | Present the characteristics of the included studies (e.g. year of publication, country, population, number of participants, data collection, methodology, analysis, research questions) | p9 and Table 1 |
| 9. Study selection results | Identify the number of studies screened and provide reasons for study exclusion (e.g. for comprehensive searching, provide numbers of studies screened and reasons for exclusion indicated in a figure/flowchart; for iterative searching describe reasons for study exclusion and inclusion based on modifications to the research question and/or contribution to theory development) | p8 and Figure 1 |
| 10. Rationale for appraisal | Describe the rationale and approach used to appraise the included studies or selected findings (e.g. assessment of conduct (validity and robustness), assessment of reporting (transparency), assessment of content and utility of the findings) | p2, p6, pp19-20, appendix 4 |
| 11. Appraisal items | State the tools, frameworks and criteria used to appraise the studies or selected findings (e.g. Existing tools: CASP, QARI, COREQ, Mays and Pope [25]; reviewer developed tools; describe the domains assessed: research team, study design, data analysis and interpretations, reporting) | pp5-6 |
| 12.Appraisal process | Indicate whether the appraisal was conducted independently by more than one reviewer and if consensus was required | p6 |
| 13.Appraisal results | Present results of the quality assessment and indicate which articles, if any, were weighted/excluded based on the assessment and give the rationale | appendix 4 summarized pp19-20 |
| 14. Data extraction | Indicate which sections of the primary studies were analyzed and how were the data extracted from the primary studies? (e.g. all text under the headings “results /conclusions” were extracted electronically and entered into a computer software) | p6 |
| 15. Software | State the computer software used, if any | p6 |
| 16. Number of reviewers | Identify who was involved in coding and analysis | p6 |
| 17. Coding | Describe the process for coding of data (e.g. line by line coding to search for concepts) | p6 |
| 18. Study comparison | Describe how were comparisons made within and across studies (e.g. subsequent studies were coded into pre-existing concepts, and new concepts were created when deemed necessary) | p6 |
| 19. Derivation of themes | Explain whether the process of deriving the themes or constructs was inductive or deductive | p6 |
| 20. Quotations | Provide quotations from the primary studies to illustrate themes/constructs, and identify whether the quotations were participant quotations of the author’s interpretation | Table 3 |
| 21. Synthesis output | Present rich, compelling and useful results that go beyond a summary of the primary studies (e.g. new interpretation, models of evidence, conceptual models, analytical framework, development of a new theory or construct) | pp15-20 |

**Supplemental Material 3: PRISMA checklist**

| **Section and Topic** | **Item #** | **Checklist item** | **Location where item is reported** |
| --- | --- | --- | --- |
| **TITLE** | | |  |
| Title | 1 | Identify the report as a systematic review. | Title, abstract, p4 “Objectives” |
| **ABSTRACT** | | |  |
| Abstract | 2 | See the PRISMA 2020 for Abstracts checklist. | As per journal requirements |
| **INTRODUCTION** | | |  |
| Rationale | 3 | Describe the rationale for the review in the context of existing knowledge. | p3 |
| Objectives | 4 | Provide an explicit statement of the objective(s) or question(s) the review addresses. | p4 |
| **METHODS** | | |  |
| Eligibility criteria | 5 | Specify the inclusion and exclusion criteria for the review and how studies were grouped for the syntheses. | p5 |
| Information sources | 6 | Specify all databases, registers, websites, organisations, reference lists and other sources searched or consulted to identify studies. Specify the date when each source was last searched or consulted. | p5 |
| Search strategy | 7 | Present the full search strategies for all databases, registers and websites, including any filters and limits used. | Appendix 1 |
| Selection process | 8 | Specify the methods used to decide whether a study met the inclusion criteria of the review, including how many reviewers screened each record and each report retrieved, whether they worked independently, and if applicable, details of automation tools used in the process. | pp5-6 |
| Data collection process | 9 | Specify the methods used to collect data from reports, including how many reviewers collected data from each report, whether they worked independently, any processes for obtaining or confirming data from study investigators, and if applicable, details of automation tools used in the process. | pp5-6 |
| Data items | 10a | List and define all outcomes for which data were sought. Specify whether all results that were compatible with each outcome domain in each study were sought (e.g. for all measures, time points, analyses), and if not, the methods used to decide which results to collect. | n/a – see ENTREQ |
|  | 10b | List and define all other variables for which data were sought (e.g. participant and intervention characteristics, funding sources). Describe any assumptions made about any missing or unclear information. | n/a – see ENTREQ |
| Study risk of bias assessment | 11 | Specify the methods used to assess risk of bias in the included studies, including details of the tool(s) used, how many reviewers assessed each study and whether they worked independently, and if applicable, details of automation tools used in the process. | Appraisal p2, p6, appendix 4 |
| Effect measures | 12 | Specify for each outcome the effect measure(s) (e.g. risk ratio, mean difference) used in the synthesis or presentation of results. | N/A |
| Synthesis methods | 13a | Describe the processes used to decide which studies were eligible for each synthesis (e.g. tabulating the study intervention characteristics and comparing against the planned groups for each synthesis (item #5)). | n/a – see ENTREQ |
|  | 13b | Describe any methods required to prepare the data for presentation or synthesis, such as handling of missing summary statistics, or data conversions. | n/a – see ENTREQ |
|  | 13c | Describe any methods used to tabulate or visually display results of individual studies and syntheses. | n/a – see ENTREQ |
|  | 13d | Describe any methods used to synthesize results and provide a rationale for the choice(s). If meta-analysis was performed, describe the model(s), method(s) to identify the presence and extent of statistical heterogeneity, and software package(s) used. | p6 |
|  | 13e | Describe any methods used to explore possible causes of heterogeneity among study results (e.g. subgroup analysis, meta-regression). | n/a – see ENTREQ |
|  | 13f | Describe any sensitivity analyses conducted to assess robustness of the synthesized results. | n/a – see ENTREQ |
| Reporting bias assessment | 14 | Describe any methods used to assess risk of bias due to missing results in a synthesis (arising from reporting biases). | Appraisal p2, p6, appendix 4 |
| Certainty assessment | 15 | Describe any methods used to assess certainty (or confidence) in the body of evidence for an outcome. | N/A |
| **RESULTS** | | |  |
| Study selection | 16a | Describe the results of the search and selection process, from the number of records identified in the search to the number of studies included in the review, ideally using a flow diagram. | p8, Figure 1 |
|  | 16b | Cite studies that might appear to meet the inclusion criteria, but which were excluded, and explain why they were excluded. | N/A |
| Study characteristics | 17 | Cite each included study and present its characteristics. | p9, Table 1 |
| Risk of bias in studies | 18 | Present assessments of risk of bias for each included study. | pp19-20, Appendix 4 |
| Results of individual studies | 19 | For all outcomes, present, for each study: (a) summary statistics for each group (where appropriate) and (b) an effect estimate and its precision (e.g. confidence/credible interval), ideally using structured tables or plots. | N/A – see ENTREQ for relevant qualitative |
| Results of syntheses | 20a | For each synthesis, briefly summarise the characteristics and risk of bias among contributing studies. |  |
|  | 20b | Present results of all statistical syntheses conducted. If meta-analysis was done, present for each the summary estimate and its precision (e.g. confidence/credible interval) and measures of statistical heterogeneity. If comparing groups, describe the direction of the effect. |  |
|  | 20c | Present results of all investigations of possible causes of heterogeneity among study results. |  |
|  | 20d | Present results of all sensitivity analyses conducted to assess the robustness of the synthesized results. |  |
| Reporting biases | 21 | Present assessments of risk of bias due to missing results (arising from reporting biases) for each synthesis assessed. |  |
| Certainty of evidence | 22 | Present assessments of certainty (or confidence) in the body of evidence for each outcome assessed. | N/A |
| **DISCUSSION** | | |  |
| Discussion | 23a | Provide a general interpretation of the results in the context of other evidence. | pp19-20 |
|  | 23b | Discuss any limitations of the evidence included in the review. | pp22-23 |
|  | 23c | Discuss any limitations of the review processes used. | p23 |
|  | 23d | Discuss implications of the results for practice, policy, and future research. | p19-22 , Table 4 |
| **OTHER INFORMATION** | | |  |
| Registration and protocol | 24a | Provide registration information for the review, including register name and registration number, or state that the review was not registered. | Cites published protocol p4 |
|  | 24b | Indicate where the review protocol can be accessed, or state that a protocol was not prepared. | p4 |
|  | 24c | Describe and explain any amendments to information provided at registration or in the protocol. | p4 |
| Support | 25 | Describe sources of financial or non-financial support for the review, and the role of the funders or sponsors in the review. | p24 |
| Competing interests | 26 | Declare any competing interests of review authors. | p24 |
| Availability of data, code and other materials | 27 | Report which of the following are publicly available and where they can be found: template data collection forms; data extracted from included studies; data used for all analyses; analytic code; any other materials used in the review. | N/A |

**Supplemental Material 4: Critical Appraisal Results**

Critical appraisal was completed using the Joanna Briggs Institute checklist,^1^ coving ten items:

Q1= Is there congruity between the stated philosophical perspective and the research methodology?

Q2= Is there congruity between the research methodology and the research question or objectives?

Q3=Is there congruity between the research methodology and the methods used to collect data?

Q4= Is there congruity between the research methodology and the representation and analysis of the data?

Q5= Is there congruity between the research methodology and the interpretation of results?

Q6=Is there a statement locating the researcher culturally or theoretically?

Q7=Is the influence of the researcher on the research, and vice- versa, addressed?

Q8= Are participants, and their voices, adequately represented?

Q9=Is the research ethical according to current criteria or, for recent studies, and is there evidence of ethical approval by an appropriate body?

Q10= Do the conclusions drawn in the research report flow from the analysis, or interpretation, of the data?

Ratings: Yes – item is present, U = Unclear if present, No – not present

| **Reference** | **Q1** | **Q2** | **Q3** | **Q4** | **Q5** | **Q6** | **Q7** | **Q8** | **Q9** | **Q10** | **Total “Yes”** |
| --- | --- | --- | --- | --- | --- | --- | --- | --- | --- | --- | --- |
| Ahmann 2013 | U | U | U | U | No | No | No | U | U | U | 0 |
| Allan 2018 | Yes | Yes | Yes | Yes | Yes | Yes | Yes | Yes | Yes | Yes | 10 |
| Armitage 2020 | Yes | Yes | Yes | Yes | Yes | Yes | Yes | Yes | Yes | Yes | 10 |
| Ay 2021 | Yes | Yes | Yes | Yes | Yes | Yes | Yes | Yes | Yes | Yes | 10 |
| Bai 2020 | Yes | Yes | Yes | Yes | U | No | No | Yes | Yes | Yes | 7 |
| Bezance 2014 | Yes | Yes | Yes | Yes | Yes | Yes | Yes | Yes | Yes | Yes | 10 |
| Budman 2022 | Yes | Yes | Yes | Yes | Yes | No | No | Yes | Yes | Yes | 8 |
| Chan 2022 | Yes | Yes | U | Yes | Yes | Yes | Yes | Yes | Yes | Yes | 9 |
| Chessell 2023 | Yes | Yes | Yes | Yes | Yes | Yes | Yes | Yes | Yes | Yes | 10 |
| Ching'oma 2022 | Yes | Yes | Yes | Yes | Yes | Yes | No | Yes | Yes | Yes | 9 |
| Cifra 2017* | U | U | Yes | U | U | No | No | U | No | Yes | 2 |
| Cottee-Lane 2017 | Yes | Yes | Yes | Yes | Yes | Yes | No | Yes | No | Yes | 8 |
| Davey 2022 | Yes | Yes | Yes | Yes | Yes | Yes | Yes | Yes | Yes | Yes | 10 |
| Eaton 2016 | Yes | Yes | Yes | Yes | Yes | No | No | Yes | Yes | Yes | 8 |
| Emerson 2019 | Yes | Yes | Yes | Yes | Yes | Yes | Yes | Yes | No | Yes | 9 |
| Harazni 2016 | Yes | Yes | Yes | Yes | Yes | No | No | Yes | Yes | Yes | 8 |
| Harden 2005 | Yes | Yes | Yes | Yes | Yes | Yes | Yes | Yes | Yes | Yes | 10 |
| Hellerova 2022 | Yes | Yes | Yes | Yes | Yes | Yes | Yes | Yes | Yes | Yes | 9 |
| Hiscock 2020 | Yes | Yes | Yes | Yes | Yes | No | No | Yes | Yes | Yes | 8 |
| Honey 2008 | Yes | Yes | Yes | Yes | Yes | No | Yes | Yes | No | Yes | 8 |
| Hunter 2021 | Yes | Yes | Yes | Yes | Yes | Yes | Yes | Yes | Yes | Yes | 10 |
| Klein 2019 | Yes | Yes | Yes | Yes | Yes | No | U | Yes | Yes | Yes | 8 |
| Konstantellou 2022 | Yes | Yes | Yes | Yes | Yes | Yes | Yes | Yes | Yes | Yes | 10 |
| Leitch 2019 | Yes | Yes | Yes | Yes | Yes | No | No | Yes | Yes | Yes | 8 |
| Long 2022 | Yes | Yes | Yes | Yes | Yes | No | Yes | Yes | Yes | Yes | 9 |
| McArdle 2019 | Yes | Yes | Yes | Yes | Yes | Yes | Yes | Yes | Yes | Yes | 10 |
| McKeague 2022 | Yes | Yes | Yes | Yes | Yes | No | No | Yes | Yes | Yes | 8 |
| Mesfin 2024 | U | Yes | Yes | Yes | Yes | No | No | Yes | Yes | Yes | 7 |
| Ott 2021 | Yes | Yes | Yes | Yes | Yes | Yes | Yes | Yes | Yes | Yes | 10 |
| Patel 2014 | Yes | Yes | Yes | Yes | Yes | No | Yes | Yes | Yes | Yes | 9 |
| Reardon 2018 | Yes | Yes | Yes | Yes | Yes | No | Yes | Yes | Yes | Yes | 9 |
| Ringer 2020 | Yes | Yes | Yes | Yes | Yes | No | Yes | Yes | Yes | Yes | 9 |
| Rosenzweig 2002 | U | Yes | Yes | Yes | U | No | No | Yes | Yes | Yes | 7 |
| Ruuskanen 2019 | Yes | Yes | Yes | Yes | Yes | U | U | Yes | Yes | Yes | 8 |
| Saulsberry 2020 | Yes | Yes | Yes | Yes | Yes | No | No | Yes | Yes | Yes | 8 |
| Sheng 2021 | Yes | Yes | Yes | Yes | Yes | No | No | Yes | Yes | Yes | 8 |
| Slowik 2004 | Yes | Yes | Yes | Yes | Yes | No | No | Yes | U | Yes | 7 |
| Sowden 2023 | Yes | Yes | Yes | Yes | Yes | No | No | No | U | U | 5 |
| Stapley 2016 | Yes | Yes | Yes | Yes | Yes | No | Yes | Yes | Yes | Yes | 9 |
| Stapley 2017 | Yes | Yes | Yes | Yes | Yes | Yes | Yes | Yes | Yes | Yes | 10 |
| Svensson 2017 | Yes | Yes | Yes | Yes | Yes | No | Yes | Yes | Yes | Yes | 9 |
| Tarver 2022 | Yes | Yes | Yes | Yes | Yes | No | No | Yes | Yes | Yes | 8 |
| Thomson 2014 | Yes | Yes | Yes | Yes | Yes | No | Yes | Yes | Yes | Yes | 9 |
| Yurdakul 2024 | Yes | Yes | Yes | Yes | Yes | Yes | Yes | Yes | Yes | Yes | 10 |
| Zhang 2023 | Yes | Yes | Yes | Yes | Yes | Yes | Yes | Yes | Yes | Yes | 10 |
| Zhang 2024 | U | Yes | Yes | Yes | Yes | No | No | Yes | Yes | Yes | 7 |

**References**

1. Lockwood C, Munn Z, Porritt K. Qualitative research synthesis: methodological guidance for systematic reviewers utilizing meta-aggregation. *International journal of evidence-based healthcare* 2015; **13**(3): 179-87.

**Supplemental Material 5: Appearance of each theme by included study**

| **First author year of publication** | **Support needs and gaps** | Support from Healthcare system | Support from school and others | Information needs | **Impact on everyday life** | **Altered family dynamics** | Conflict with CYP | Strain in other relationship | Negotiating a new role | **Parental worries and fears** | **Emotional experience of caregivers** | Frustration, exhaustion, stress and sorrow | Self-conscious emotions | **Self-care paradox** |
| --- | --- | --- | --- | --- | --- | --- | --- | --- | --- | --- | --- | --- | --- | --- |
| Ahmann 2013 |  | X | X |  |  |  |  |  |  |  |  | X | X |  |
| Allan 2018 |  | X |  |  | X |  |  |  |  |  |  |  |  | X |
| Armitage 2020 |  |  | X |  | X |  | X | X |  | X |  | X | X | X |
| Ay 2021 |  | X |  | X |  |  | X |  |  |  |  | X |  |  |
| Bai 2020 |  |  |  | X | X |  | X | X |  | X |  | X | X |  |
| Bezance 2014 |  | X |  |  | X |  |  |  | X | X |  | X | X | X |
| Budman 2022 |  | X | X | X |  |  | X | X | X | X |  |  | X | X |
| Chan 2022 |  |  | X |  |  |  |  |  | X |  |  |  | X | X |
| Chessell 2023 |  | X | X |  | X |  | X | X | X | X |  | X | X | X |
| Ching'oma 2022 |  | X | X | X | X |  | X |  |  | X |  | X | X | X |
| Cifra 2017* |  | X |  | X | X |  |  |  |  |  |  |  | X |  |
| Cottee-Lane 2017 |  | X |  | X |  |  |  |  | X |  |  | X |  | X |
| Davey 2022 |  | X | X | X |  |  |  |  |  | X |  |  |  | X |
| Eaton 2016 |  |  |  |  | X |  |  |  |  |  |  |  | X |  |
| Emerson 2019 |  |  |  |  | X |  | X | X |  |  |  | X | X | X |
| Harazni 2016 |  |  | X |  | X |  | X | X | X | X |  | X | X | X |
| Harden 2005 |  | X |  | X | X |  |  |  | X |  |  | X | X |  |
| Hellerova 2022 |  |  |  | X | X |  |  |  |  |  |  | X | X |  |
| Hiscock 2020 |  |  |  |  | X |  |  |  | X | X |  | X |  |  |
| Honey 2008 |  | X |  | X |  |  |  |  |  |  |  | X |  |  |
| Hunter 2021 |  |  | X |  |  |  |  | X | X | X |  |  | X |  |
| Klein 2019 |  | X |  | X |  |  |  |  |  |  |  |  |  | X |
| Konstantellou 2022 |  | X | X |  |  |  |  |  | X |  |  | X | X | X |
| Leitch 2019 |  |  | X |  |  |  | X | X | X |  |  |  | X | X |
| Long 2022 |  |  | X |  |  |  |  | X |  |  |  | X | X | X |
| McArdle 2019 |  | X | X |  | X |  |  |  |  | X |  |  | X |  |
| McKeague 2022 |  |  | X |  |  |  |  |  | X | X |  |  | X | X |
| Mesfin 2024 |  |  |  |  | X |  |  |  | X | X |  | X | X | X |
| Ott 2021 |  |  | X |  |  |  |  | X |  | X |  |  |  | X |
| Patel 2014 |  | X | X | X | X |  |  | X | X | X |  | X | X | X |
| Reardon 2018 |  | X | X | X | X |  |  | X | X | X |  |  | X |  |
| Ringer 2020 |  |  | X |  | X |  | X |  |  |  |  |  | X | X |
| Rosenzweig 2002 |  |  | X |  | X |  |  |  |  |  |  |  |  |  |
| Ruuskanen 2019 |  |  |  | X | X |  | X | X |  |  |  |  |  | X |
| Saulsberry 2020 |  | X |  |  |  |  | X |  | X |  |  | X |  |  |
| Sheng 2021 |  | X |  |  | X |  |  |  |  | X |  | X | X | X |
| Slowik 2004 |  | X | X |  | X |  | X | X | X | X |  |  | X | X |
| Sowden 2023 |  | X | X | X | X |  |  | X | X |  |  | X |  | X |
| Stapley 2016 |  | X | X | X |  |  | X |  | X |  |  | X |  | X |
| Stapley 2017 |  |  | X | X | X |  | X | X | X | X |  | X |  |  |
| Svensson 2017 |  |  | X | X | X |  |  | X | X | X |  | X | X | X |
| Tarver 2022 |  | X |  | X | X |  | X |  |  |  |  |  |  | X |
| Thomson 2014 |  | X | X | X |  |  |  | X | X |  |  |  | X | X |
| Yurdakul 2024 |  |  | X |  | X |  |  |  |  | X |  | X |  |  |
| Zhang 2023 |  | X | X |  | X |  |  | X |  |  |  |  | X | X |
| Zhang 2024 |  |  |  |  | X |  |  |  |  | X |  | X | X |  |
